# A Review on Recent Computational Methods for Predicting Noncoding RNAs

**DOI:** 10.1155/2017/9139504

**Published:** 2017-05-03

**Authors:** Yi Zhang, Haiyun Huang, Dahan Zhang, Jing Qiu, Jiasheng Yang, Kejing Wang, Lijuan Zhu, Jingjing Fan, Jialiang Yang

**Affiliations:** ^1^Department of Mathematics and Information Retrieval of Library and Hebei Laboratory of Pharmaceutic Molecular Chemistry, Hebei University of Science and Technology, Shijiazhuang, Hebei 050018, China; ^2^College of Life Science and Technology, Huazhong Agricultural University, Wuhan, Hubei 430070, China; ^3^Department of Network Engineering, School of Information Science and Engineering, Hebei University of Science and Technology, Shijiazhuang 050018, China; ^4^Department of Civil and Environmental Engineering, National University of Singapore, Singapore 117576; ^5^School of Mathematics and Information Science, Henan Polytechnic University, Henan 454000, China

## Abstract

Noncoding RNAs (ncRNAs) play important roles in various cellular activities and diseases. In this paper, we presented a comprehensive review on computational methods for ncRNA prediction, which are generally grouped into four categories: (1) homology-based methods, that is, comparative methods involving evolutionarily conserved RNA sequences and structures, (2) de novo methods using RNA sequence and structure features, (3) transcriptional sequencing and assembling based methods, that is, methods designed for single and pair-ended reads generated from next-generation RNA sequencing, and (4) RNA family specific methods, for example, methods specific for microRNAs and long noncoding RNAs. In the end, we summarized the advantages and limitations of these methods and pointed out a few possible future directions for ncRNA prediction. In conclusion, many computational methods have been demonstrated to be effective in predicting ncRNAs for further experimental validation. They are critical in reducing the huge number of potential ncRNAs and pointing the community to high confidence candidates. In the future, high efficient mapping technology and more intrinsic sequence features (e.g., motif and *k*-mer frequencies) and structure features (e.g., minimum free energy, conserved stem-loop, or graph structures) are suggested to be combined with the next- and third-generation sequencing platforms to improve ncRNA prediction.

## 1. Background

A noncoding RNA (ncRNA) is a functional RNA that is transcribed from a DNA but does not encode a protein. According to transcriptomic and bioinformatics studies, there are thousands of ncRNAs classified into different categories based on their functions and lengths including transfer RNA (tRNA), ribosomal RNA (rRNA), microRNA (miRNA), and long ncRNA (lncRNA) to name a few [1–3].

These ncRNAs play important roles in various cellular processes. For example, rRNA catalyzes the peptide bond formation between amino acids in translation process [4], miRNA is important in transcription process and performs posttranscriptional regulation of gene expression [5], and lncRNA plays critical diverse roles in X inactivation, imprinting, and regulation of epigenetic marks and gene expression [6–8]. In addition, they also exhibit enormous importance in connection with various diseases. For example, the miR-17-92 cluster functions as oncogenes while the miR-15a–miR-16-1 cluster functions as tumour suppressors [9].* ANRIL*, one type of lncRNA, is related to coronary disease, type II diabetes, and intracranial aneurysm [10]. The readers are referred to a review by Esteller [11] and Chen et al. [12] for more information about specific correlations between ncRNAs and human diseases. Specifically, Esteller [11] provides a review on the relationship between dysfunctions of ncRNAs including miRNA, PIWI-interacting RNAs (piRNAs), small nucleolar RNAs (snoRNAs), transcribed ultraconserved regions (T-UCRs), and large intergenic noncoding RNAs (lincRNAs) and a few diseases including tumorigenesis and neurological, cardiovascular, developmental, and other diseases. Chen et al. [12] discussed the roles of lncRNAs in critical biological processes and human diseases like various cancers, diabetes, and AIDS.

Due to the important roles of ncRNAs in cellular processes and disease development, many experimental and bioinformatics methods have been developed to predict ncRNAs and their functions. As for experimental methods, enzymatic and chemical RNA sequencing, parallel cloning of ncRNAs by specialized cDNA libraries, microarray analysis, and genomic SELEX are among the most popular ones. The readers are referred to a review paper for the details of these methods [13]. However, the experimental methods are expensive and time-consuming, and thus hundreds of computational methods have also been developed to prioritize highly confident ncRNA candidates for further experimental validation. In this paper, we present a comprehensive review on these computational methods. We are fully aware that there have already been several review articles on this hot topic [14–17]. However, they either focus on a specific ncRNA category or have been outdated and could not present a panoramic view of the field.

## 2. Main Text

**Figure 1:**
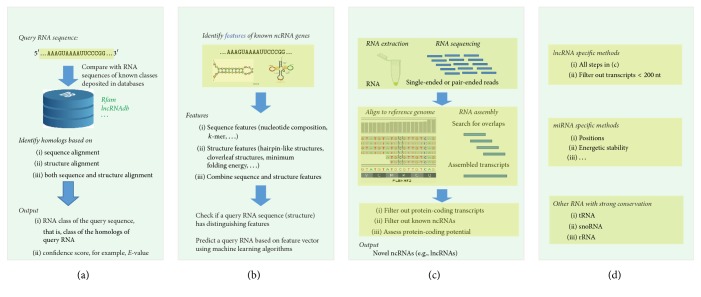
Four popular categories of computational methods in predicating ncRNAs. (a) Homology-based methods, which compare a query RNA with known ncRNAs deposited in databases based on sequence or structure alignment; (b) de novo methods, which predict ncRNA from primary sequences or structure based on general principles that govern ncRNA folding or statistical tendencies of *k*-mer features; (c) transcriptional sequencing and assembling based methods, which utilize next-generation sequencing and transcriptome data; and (d) RNA family specific methods, which predict specific ncRNA classes.

Generally speaking, there are three major categories of computational methods in predicting ncRNAs, namely, (1) homology-based methods involving evolutionarily conserved RNA sequences and structures, (2) de novo methods using RNA sequence and structure features, and (3) transcriptional sequencing and assembling based methods, according to chronological order of their occurrences. Since miRNA and lncRNA have very specific methods, we reviewed them separately and called these methods RNA family specific methods (Figure 1).

### 2.1. Homology-Based Methods

As probably the earliest ncRNA prediction methods, homology-based methods assume that sequence or structure similar RNAs are evolved from a common ancestor and thus share function similarities [18, 19]. Given a query RNA, these methods usually compare it with known ncRNAs deposited in databases based on sequence or structure alignment. The RNA is predicted to be in a specific ncRNA family if it has sufficient similarity with known ncRNAs in that family (Figure 1(a)). There are a number of ncRNA databases. For example, 2,474 structural families of ncRNAs were cataloged in the database Rfam (version 12.1, April 2016) [20]. We listed a few popular homology-based methods in Table 1, which are further classified into sequence-based methods, structure-based methods, and hybrid methods.

#### 2.1.1. Sequence-Based Methods

These methods rely purely on sequence conservations inferred by alignment methods like BLAST [18] and BLAT [21]. They first identify short (gapped) matches called seeds [22] between the query ncRNA and any ncRNA in the database, which are then expanded in both directions to form high-scoring segment pair (HSPs). The statistical significance of a HSP or the joining of several HSPs is evaluated by expected value (called *E*-value). The query ncRNA is classified into the family containing the ncRNA with the lowest *E*-value.

#### 2.1.2. Structure-Based Methods

Sequence-based methods are usually very fast. However, it is commonly believed that ncRNAs are less conserved in sequence. Thus, another category of homology-based methods is introduced based on structure conservations. Instead of sequence alignment, these methods use RNA secondary structure alignment to measure RNA similarity. Popular methods include QRNA [19] and RNAz [23]. Specifically, QRNA compares query RNA with known RNAs using “three probabilistic pair-grammars: a pair stochastic context-free grammar modeling alignments constrained by structural RNA evolution, a pair hidden Markov model modeling alignments constrained by coding sequence evolution, and a pair hidden Markov model modeling a null hypothesis of position-independent evolution” [19], whereas RNAz compares RNAs based on conserved secondary structure and thermodynamic stability [23].

#### 2.1.3. Hybrid Methods

A more robust RNA similarity measure was obtained by incorporating both sequence and structure information. For example, Infernal [24] uses covariance models, which score a combination of sequence consensus and RNA secondary structure consensus to predict ncRNAs homologous to ncRNA families in Rfam [20, 24]. MASTR [25] makes use of simulated annealing method to perform sequence alignment and structural alignment simultaneously.

Though homology-based methods have been extensively used due to their advantages in speed, however, they have a few limitations. First, they compare the query RNA with known ncRNA families and thus are incapable of predicting new ncRNA families. Second, they rely on sequence or structure conservations and thus are inapplicable to predict ncRNAs lacking conservation in sequence and structure. As a result, de novo methods are proposed to solve such dilemma.

### 2.2. De Novo Methods Using RNA Sequence and Structure Features

Unlike homology methods which require the information of RNAs similar (or homologous) to the query RNA, de novo methods predict ncRNA from primary sequences or structure based on general principles that govern ncRNA folding energetics and/or statistical tendencies of *k*-mer features that native ncRNA sequences and structures acquire (Figure 1(b)). Based on the source of common features, de novo methods can be divided into sequence feature based methods which only use sequence features, structure feature methods, and hybrid feature methods which use both features.

#### 2.2.1. Sequence Feature Based Methods

One important feature for sequence-based de novo methods is nucleotide composition, which applies for identifying ncRNAs in species with nucleotide compositional biases. For example, by calculating the GC content, Wang et al. identified ncRNA genes with stable secondary structure in an AT-rich extreme hyperthermophile [26]. Another commonly used nucleotide composition is *k*-mer (nucleotide sequence of length *k*) frequencies. Methods in this category exploit the finding that the frequencies of many *k*-mers for ncRNAs in a specific family usually share similar probability distribution. Thus, new ncRNAs can be predicted based on the distribution of their *k*-mer frequencies. For example, Panwar et al. used the trinucleotide composition (i.e., 3-mer) to predict ncRNA by a support vector machine (SVM) based algorithm [27]. Sun et al. proposed Coding-Non-Coding Index (CNCI), by profiling adjoining nucleotide triplets (i.e., 6-mer) to effectively distinguish protein-coding and noncoding sequences independent of known annotations [28]. In addition, Li et al. developed an algorithm named PLEK to discriminate lncRNAs from mRNAs based on a combination of 1 to 5 mers [29].

Since a single type of sequence feature might be insufficient in effectively identifying ncRNAs, other features have also been proposed in conjunction with nucleotide composition. We summarized a few popular sequence feature based de novo ncRNA identification methods in Table 2. For example, CONC [30] incorporates a few types of features including sequence length, nucleotide composition, and reading frame to characterize ncRNAs. CPC [31] combines the longest reading frame in the three forward frames, log-odds score, coverage of the predicted ORF, and integrity of the predicted ORF, to identify ncRNAs.

#### 2.2.2. Structure Feature Based Methods

The secondary structures of some kinds of functional RNA are more conserved than their primary sequences [32]. For example, miRNA precursors share common hairpin-like structures and tRNAs share cloverleaf structures. The structure with (or around) the minimum folding energy (MFE) is usually regarded as the most possible fold structure of an RNA. Thus, MFE is extensively used to predict secondary structure of ncRNA sequences. Popular MFE-based methods include RNAfold [33], Mfold [34], and Afold [35]. RNAfold calculates MFE by assigning free energies to both loops and stems, whereas Mfold only assigns free energies to loops. Afold improves the speed in evaluating all possible internal loops by an algorithm constructing sets of conditionally optimal multibranch loop free (MLF) structures. However, it is generally insufficient to use MFE alone for the detection of ncRNAs since different secondary structures of a given RNA sequence may have very similar MFE [36]. As a result, more structure features like thermodynamic stability are also employed in predicting ncRNA [37].

#### 2.2.3. Hybrid Feature Based Methods

As a trend, more and more de novo methods tend to combine both RNA sequence and RNA structure to improve the sensitivity and specificity in predicting ncRNAs.

**Figure 2:**
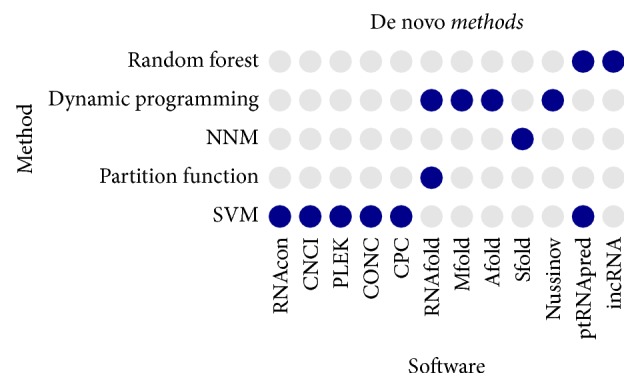
Popular de novo methods and the statistical algorithms applied.

For example, Gupta et al. developed a new algorithm ptRNApred to identify and classify posttranscriptional RNA with dinucleotide properties of sequence and secondary structure feature, for example, numbers of loops, bulges, and hairpins or the frequency of nucleotides involved in substructures [45]. It can predict ptRNA-subclasses in eukaryotes including snRNA, snoRNA, RNase P, RNase MRP, Y RNA, and telomerase RNA. We summarized popular de novo ncRNA prediction methods using RNA sequence and structure features in Table 3. For a better view, we also plotted some popular de novo methods and their prediction algorithms in Figure 2. Support vector machine (SVM) is probably the most frequently used method for de novo ncRNA prediction.

De novo methods are capable of predicting new ncRNA families and classifying ncRNAs lacking conservation with existing ones. They usually have higher sensitivity and lower specificity than homology-based methods. However, this kind of methods depends largely on the features extracted. With the enrichment of biological, chemical, and dynamic knowledge of ncRNA, there might be some further informative features to be extracted, which will greatly benefit de novo ncRNA prediction [46, 68].

### 2.3. Transcriptional Sequencing and Assembling Based Methods

More recently, with the advances in next-generation sequencing (NGS), especially RNA sequencing (RNA-seq) techniques, more and more transcriptome data are available, which have been utilized to discover novel ncRNAs. A general workflow of transcriptional sequencing and assembling based ncRNA prediction method is described in Figure 1(c). Different from homology-based and de novo methods which require specific RNA sequences, methods in this category usually start from raw single-ended or pair-ended reads. The reads are then mapped into a reference genome and the mapped reads are assembled into transcripts based on overlapping information. After removing protein-coding RNA and known ncRNA transcripts, the remaining transcripts are further assessed for protein-coding potential and novel ncRNAs are reported if the potential is low.

In practice, RNA-seq data are usually combined with other features and methods including tilling array [47], graph-kernel SVM [49], structure features and common motifs [69], differential gene expression (DGE) data [48], and exon array [50] to predict specific ncRNAs. For example, tiling array [47] is used to scan the long and macro non-protein-coding RNAs related to cell-cycle, p53, and STAT3 pathways. DGE is used for discovering novel polyA+noncoding transcripts within human genome [48]. BlockClust [49] tries to predict the ncRNA modified after its transcription by combining the sequence and secondary structure information with a graph-kernel SVM, whose novel thinking lies in a new strategy to formulate expression profiles in compact discrete structures using fast graph-kernel techniques. We summarized some popular sequencing and assembling based ncRNA predication algorithms in Table 4.

As an advantage over homology-based methods and de novo methods, RNA-seq based methods can directly sequence coding and noncoding RNA transcripts with high sensitivity and low false positive rate. It can especially detect new scripts and alternative splicing. However, sometimes it is difficult to tell ncRNAs from protein-coding RNAs and thus other features like sequence conservation [53], deciphering abstract graphical representation [49], designing exon probes [50], finer terminal stem-loop feature [51], or *k*-mer frequency [52] are often utilized together with RNA-seq analysis to infer ncRNAs. In this sense, one may regard the RNA-seq technology as a platform rather than a certain method.

### 2.4. RNA Family Specific Methods

Since miRNA and lncRNA are two special and important ncRNAs, we reviewed a few computational methods related to them separately (Figure 1(d)).

#### 2.4.1. miRNA Specific Methods

miRNAs are very short in length, usually around 22 nt. The short length and relatively low conservation of pre-miRNA sequences restrict the usage of sequence-based methods in identifying miRNAs. Fortunately, it is known that miRNAs are mostly derived from regions of RNA transcripts that fold back on themselves to form short hairpins, which make this RNA relatively conserved in secondary structure. Thus, a few methods exploit more secondary features for new miRNA gene detection instances. For example, as a homology-based method, miRAlign employs sequence alignment, secondary structure alignment, and miRNA's position on the stem-loop structure to identify RNA homologs. It has higher sensitivity and comparable specificity than other homology-based methods [70]. MiPred adopts the local contiguous structure sequence composition, MFE, and *P* value of randomization test to predict miRNA precursor with a random forest algorithm [54]. We summarized popular methods for predicting miRNA in Table 5.

#### 2.4.2. lncRNA Specific Methods

Long noncoding RNAs (lncRNAs) are ncRNAs longer than 200 nt, including long intronic noncoding RNA and intergenic noncoding RNA. lncRNAs are believed to regulate gene expression through changing chromatin state and correlate with cancer pathogenesis and various clinical traits [63–66, 71]. In fact, lncRNA prediction is a very challenging task, because many lncRNAs exhibit low sequence and structure conservation; moreover, they are often capped and spliced. Some databases like lncRNAdb [72] provide comprehensive annotations of specific lncRNAs, for example, eukaryotic lncRNAs. A general flow to identify lncRNA is as follows: first the transcriptome data are annotated and the protein-coding sequences are filtered; then sequences shorter than 200 nt are removed and the remaining ones are viewed as candidate lncRNAs [63]; finally, the candidate lncRNAs are evaluated based on features like secondary structures [73, 74], protein-coding ability [28, 29], conserved splicing sites [75], DGE+RNA-seq, conserved promoters [66], and chromatin signatures such as “K4–K36” domain [67], and only those that pass certain significance levels are inferred to be lncRNAs. We summarized popular lncRNA prediction methods in Table 6.

Besides the above two RNA families, some specific classification and prediction methods have been developed for ncRNAs with strong conservation information, for example, tRNA [76–78], snoRNA [79–81], and rRNA [82]. Recently, the largest ncRNA set, piRNA, can be predicted by an improved Fisher algorithm with 1364-D vectors representing RNA sequences [83, 84].

## 3. Conclusions

It is very important to predict ncRNAs since they are related to many diseases [85, 86]. Many ncRNA sequences are stored in databases such as fRNAbd [87], NONCODE [88], and Rfam [20] and grouped into classes based on their structures. The popular software Infernal [24] can predict 2,474 families of ncRNA. However, there are still ncRNAs that cannot be predicted by Infernal, including piRNA, Air, BC200, mature miRNA, gRNA, mRNA-like RNA, BC1 RNA, BM1 RNA, and so on. The major issue is that these ncRNAs lack sequence and structure conservation. To thoroughly predict the ncRNA classes and whole ncRNA set, we need to construct a series of new methods, including extracting new features and developing novel algorithms.

Homology search has become much faster with the development of bioinformatics tools, for example, from Smith-Waterman dynamic programming algorithm to BLAST or GMAP [89] based on simplified consecutive *k*-mer match or gapped *k*-mer (also called spaced seeds) techniques [22, 90]. However, these methods are less sensitive in ncRNA identification. On the other hand, de novo algorithms try to retrieve significant intrinsic features from RNA sequences, structures, energy, stability, and even deep-sequencing mapping profile. They use the features to discriminate a certain class of ncRNAs from other RNA sequences. However, de novo algorithms have high false positive rate. At present, how to combine these features and select a proper classifying machine is another hotspot to improve the sensitivity and specificity of ncRNA identification. With the rapid increasing of second- and third-generation sequencing (TGS) data, the information derived from deep-sequencing and single-molecule long-read sequencing may provide a great opportunity to enhance the efficiency in ncRNA prediction.

In addition, it has become central for understanding biological process by studying RNA globally. However, methods like microarrays and short-read sequencing are incapable of describing the entire RNA molecule from 5′ to 3′ end. Scientists use single-molecule long-read sequencing technology from Pacific Biosciences to sequence the polyadenylated RNA complement for human, without the need for fragmentation or amplification [91]. TGS can get full-length RNA molecules of up to 1.5 kb with little sequence loss at the 5′ ends. In total, ~14,000 spliced GENCODE genes of human were identified [91], but >10% of the alignments are mapped to unannotated regions; these transcripts are novel noncoding RNAs. Obviously, TGS may give more power to lncRNA discovery.

Finally, in order to assemble and correct long transcripts, one can integrate reads sequenced by five sequencing platforms including Illumina HiSeq, Life Technologies' PGM and Proton, Pacific Biosciences RS, and Roche's 454 [92]. Software programs like TMAP (PGM and Proton), GSRM (454), and GMAP (PacBio) are the best in mapping the sequencing reads to a reference genome. It has been shown that the integration results showed high concordance in both intraplatform and interplatform studies [92]. In addition, the integrated data also performed effectively in analyzing degraded RNA samples. Thus, platform integration is very promising for improvement of RNA-seq as well as ncRNA identification in the future.

## Figures and Tables

**Table 1:** Homology-based ncRNA function prediction methods.

Name	URL	Feature	Prediction algorithm
BLAST [18]	https://blast.ncbi.nlm.nih.gov/Blast.cgi	Sequence only	BLAST *E*-value

BLAT [21]	https://genome.ucsc.edu/cgi-bin/hgBlat	Sequence only	Pairwise alignment algorithm

CSHMM [38]		Structure only	A discriminant function based on likelihood score for a hidden Markov model (CSHMM)

Infernal [20, 24]	http://infernal.janelia.org/	Sequence and RNA secondary structure	Stochastic context-free grammars called covariance models (CMs), HMM

ERPIN [39]	http://rna.igmors.u-psud.fr/Software/erpin.php	Sequence and RNA secondary structure	Profile-based dynamic programming algorithm and *E*-value

QRNA [19]		Sequence only	Pair hidden Markov model

RNAz [23]	https://www.tbi.univie.ac.at/~wash/RNAz/	RNA secondary structure and thermodynamic stability	Support vector machine regression

Evofold [40]	https://github.com/bowhan/kent/blob/master/src/hg/makeDb/trackDb/drosophila/evofold.html	A log-odds score	Phylogenetic stochastic context-free grammars

MASTR [25]	http://mastr.binf.ku.dk/	Mutual information with gap penalty, six canonical base pairs, stacking of adjacent base pairs, and the score combining the log-likelihood of the alignment, a covariation term, and the base-pair probabilities	Sampling approach by Markov chain Monte Carlo in a simulated annealing framework

**Table 2:** De novo ncRNA function prediction methods using RNA sequence features.

Name	URL	Feature	Prediction algorithm
RNA*con *[27]	http://crdd.osdd.net/raghava/rnacon/	3-mer of nucleotides	SVM with parameters and kernels optimized by model training

CNCI [28]	https://github.com/www-bioinfo-org/CNCI	Frequency of adjoining nucleotide triplets (6-mer), the length and *S*-score of most-like CDS, length-percentage, score-distance, and codon-bias	SVM using the standard radial basis function kernel

PLEK [29]	https://sourceforge.net/projects/plek/	Normalized frequencies of 1–5 mers of RNA sequences	SVM

CONC [30]		Peptide length, amino acid composition, nucleotide frequencies, predicted secondary structure content, predicted percentage of exposed residues, compositional entropy, number of homologs from database searches, and alignment entropy	SVM

CPC [31]	http://cpc.cbi.pku.edu.cn/	The longest reading frame in the three forward frames, log-odds score, coverage of the predicted ORF, and integrity of the predicted ORF	SVM

**Table 3:** De novo ncRNA prediction methods using RNA structure features.

Name	URL	Feature	Prediction algorithm
RNAfold [33]		Base-pair probabilities and MFE	Partition function and dynamic programming

Mfold [34]	http://unafold.rna.albany.edu/?q=mfold	MFE	Dynamic programming

Afold [35]	ftp://ftp.ncbi.nlm.nih.gov/pub/ogurtsov/Afold	Sets of conditionally optimal multibranch loop free structures	Dynamic programming

Sfold [41]	http://sfold.wadsworth.org/cgi-bin/index.pl	Internal loops, sets of conditionally optimal MLF structures	Nearest-neighbour model (NNM)

Nussinov [42]	http://www.pnas.org/content/77/11/6309	Individual base pairs and loop structure with the lowest free energy	Dynamic programming

Partition function method [43]	http://www.ncbi.nlm.nih.gov/pubmed/1695107	Full equilibrium partition for secondary structure and the probabilities of various substructures	Dynamic programming

Zhang [44]	http://www.ncbi.nlm.nih.gov/pubmed/16395542/	MFE and GC content	Dynamic programming

ptRNApred [45]	http://www.ptrnapred.org/	91 features including (1) 7 selected dinucleotide properties as well as their dinucleotide values, (2) 52 properties derived from the secondary structure, for example, the number of loops, and (3) 32 triplet element properties	Random forest and SVM

incRNA [46]	http://incrna.gersteinlab.org/	9 genomic features including 4 expression features, 3 sequence information, and 2 RNA structure features	Random forest

**Table 4:** Sequencing-assembling based whole ncRNA set methods.

Name	URL	Feature	Prediction algorithm
Tilling array [47]	http://www.genomebiology.com/2014/15/3/R48	Synonymous amino acid substitutions, reading frame conservation, and the occurrence of premature stop codons	RNAcode algorithm and biweight kernels

DigitagCT [48]	http://cractools.gforge.inria.fr/softwares/digitagct	Genomic sequences, DGE tags, and tiling array expression	Infernal and BLASTN

BlockClust [49]	http://toolshed.g2.bx.psu.edu/view/rnateam/blockclust_workflow	(1) The block group: entropy of read starts, entropy of read ends, entropy of read lengths, median of normalized read expressions and normalized read expression levels in first quantile; (2) block: number of multimapped reads, entropy of read lengths, entropy of read expressions, minimum read length and block length, and (3) block edge: contiguity and difference in median read expressions	Graph-kernel SVM

Noncoder [50]	http://noncoder.mpi-bn.mpg.de/	Sequence homology, evolutionary information, the longest reading frame in three forward frames, log-odds score, coverage of the predicted orf, and integrity of the predicted orf	BLAT and PhyloCSF

Vicinal [51]	http://nar.oxfordjournals.org/content/42/9/e79.full.pdf+html	Chimeric RNA-cDNA fragments and terminal stem-loop	Bowtie 2 local mapping, filtering, and Vicinal mapping

CoRAL [52]	http://nar.oxfordjournals.org/content/41/14/e137.full.pdf+html	Read length, abundance of antisense transcription, 5′ and 3′ positional entropy, four nucleotide frequencies transformed into a log-odds ratio relative to equal base frequencies, and MFE	Multiclass classification random forest

FlaiMapper [53]	http://www.ncbi.nlm.nih.gov/pubmed/25338717	Densities of start and end positions of aligned reads and read lengths	Peak detection on the start and end position densities followed by filtering and a reconstruction process

**Table 5:** Methods to predict miRNA.

Name	URL	Feature	Prediction algorithm
CSHMM [38]		Structure only	A discriminant function based on likelihood score for a hidden Markov model

MiPred [54]		32 possible combinations of the middle nucleotide among the triplet elements, local contiguous structure sequence composition, MFE, and *P* value of randomization test	Random forest

PlantMiRNAPred [55]	http://nclab.hit.edu.cn/PlantMiRNAPred/	115 features including (1) 17 primary sequence-related features, (2) 64 secondary structure-related features, and (3) 34 energy- and thermodynamics-related features	SVM

miRdentify [56]	http://www.ncrnalab.dk/#mirdentify/mirdentify.php	5′ heterogeneity, overhangs, negative numbers indicating 5′ overhang, thermodynamics, entropy, tailing, and multimapping	Mapping and seeking duplex-forming reads within 46-80nt distance with the guide strand

CID-miRNA [57]	https://github.com/alito/CID-miRNA	Secondary structure likelihood	Stochastic context-free grammar model, Chomsky normal form; Cocke-Young-Kasami algorithm, and Classification tree

miRank [58]	https://omictools.com/mirank-tool	36 global and local intrinsic features, including the normalized MFE of folding, the normalized base pairing propensities of both arms, and the normalized loop length	Belief propagation on a weighted graph, random walks-based ranking algorithm

miRCat [59]	http://srna-workbench.cmp.uea.ac.uk/tools/analysis-tools/mircat/	*E*-value of alignment and MFE of secondary structure	Dynamic programming

mirTool [60]	http://centre.bioinformatics.zj.cn/mirtools/	miRNA/miRNA, absolute/relative reads count, and the most abundant tag	Folding the flanking genomic sequence using the miRDeep program

miRanalyzer [61]	http://bioinfo5.ugr.es/miRanalyzer/miRanalyzer.php	Number of bindings in read cluster sequence, normalized mean free energy of precursor sequence, number of bindings in precursor, length of read cluster, the corresponding putative mature star sequence, number of bindings in read cluster divided by the read cluster length, number of reads in read cluster, mean free energy of precursor sequence, degree of bulb asymmetry in precursor, and the number of bulbs in precursor secondary structure	Random forest

sRNAbench [62]	http://bioinfo5.ugr.es/sRNAbench/	Within cluster ratio, 5′ fluctuations, most frequent to all ratio, minimum number of hairpin bindings, minimum number of mature bindings, most frequent read, length interval, and minimum reads	Hierarchical clustering

**Table 6:** Methods to predict lncRNAs.

Name	Feature	Prediction algorithm
Estimating lincRNome size for human [63]	lincRNA numbers validated experimentally in human and mouse, and their overlap lincRNA number	System of nonlinear equations

Classifying human lncRNA [64]	RNA sequence-structure patterns (RSSPs) describing 42 highly structured families, motif binding sites extracted as 1314 Position-Weight Matrices (PWMs), all *k*-words of length *k* = 2,3, 4,5, 6,7, 8, the sequence complexity	Classifying human lncRNA by being able (or disable) to bind the polycomb repressive complex (PRC2), SVM with linear kernel

Identify, classify, and localize maize lncRNAs [65]	Transcript length, open reading frame (ORF) size, and homology with known proteins	SVM

The GENCODE v7 catalog of human lncRNA [66]	Lack of homology with known proteins, no reasonable-sized open reading frame (ORF), and no high conservation, confirmed by PhyloCSF through the majority of exons conserved promoters	Manual annotation and pattern recognition

Highly conserved large noncoding RNAs [67]	Chromatin signatures “K4–K36” domain	Maximum CSF score observed across the entire genomic locus

## Abbreviations

ncRNA: Noncoding RNA
RT-PCR: Reverse transcription polymerase chain reaction
tRNA: Transfer RNA
rRNA: Ribosomal RNA
snoRNA: Small nucleolar RNA
miRNA: microRNA
siRNA: Small interfering RNA
snRNA: Small nuclear RNA
exRNA: Extracellular RNA
piRNA: Piwi-interacting RNA
lncRNA: Long noncoding RNA
Xist: X-inactive specific transcript
HOTAIR: HOX transcript antisense RNA
ceRNA: Competing endogenous RNA
MALAT-1: Metastasis-associated lung adenocarcinoma transcript 1
HSP: High-scoring segment pair
HMM: Hidden Markov model
MFE: Minimum folding energy
SVM: Support vector machine
DGE: Differential gene expression.
